# Supplementary figures and images for: Spawning aggregations of checkered snapper (Lutjanus decussatus) and blackspot snapper (L. fulviflamma): seasonality, lunar-phase periodicity and spatial distribution within spawning ground
Source: PeerJ. 2023 Sep 11;11:e15991. doi: 10.7717/peerj.15991 (PMC10710171; doi:10.7717/peerj.15991)

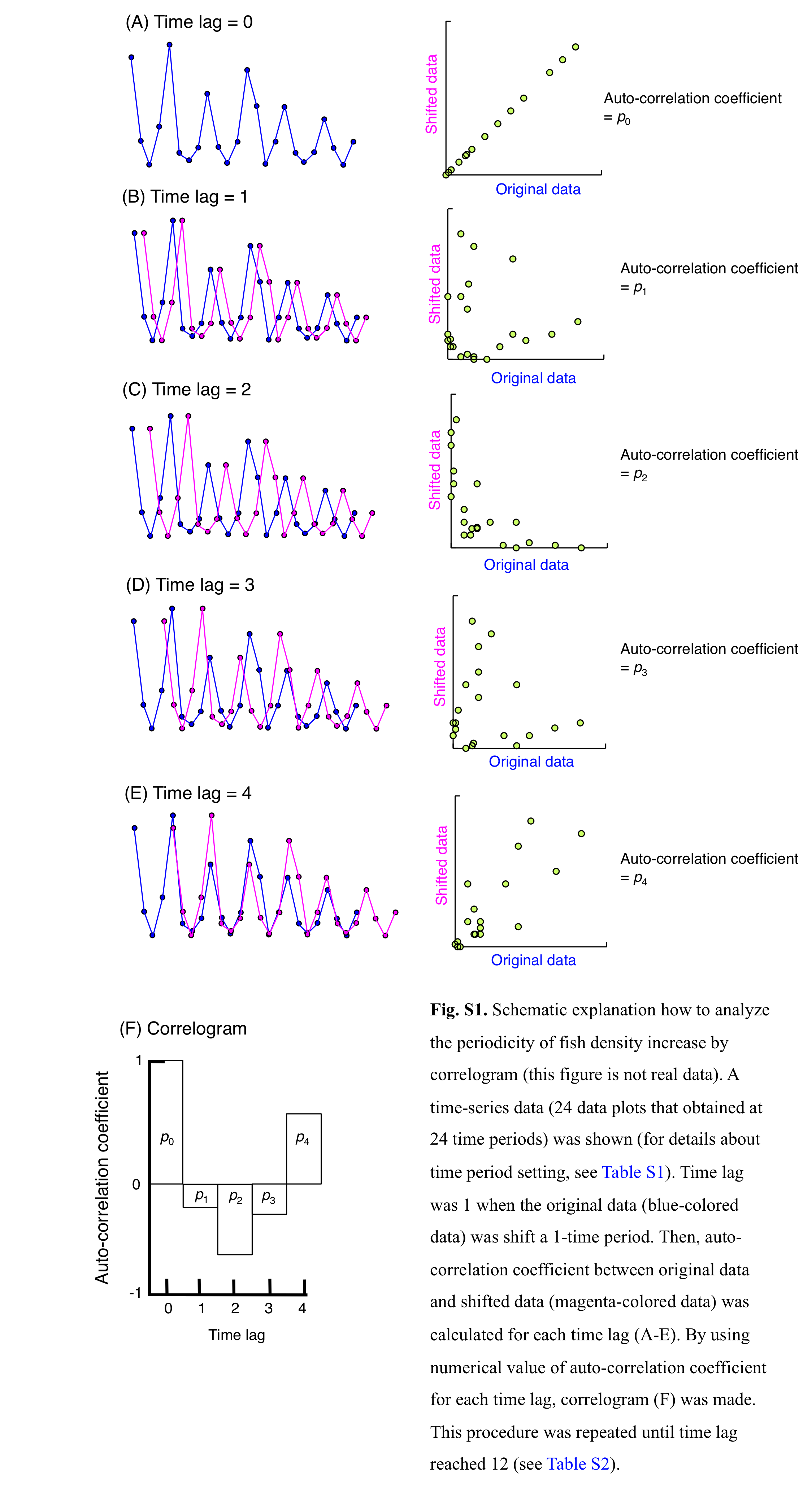

Supplement: Supplemental Information 7 — A time-series of data (24 data plots that were obtained at 24 time periods) is shown (for details about time period setting, see Table S1). Time lag was 1 when the original data (blue-colored data) was shift a 1-time period. Then, auto-correlation coefficient between original data and shifted data (magenta-colored data) was calculated for each time lag (A–E). By using numerical value of the auto-correlation coefficient for each time lag, correlogram (F) was made. This procedure was repeated until time lag reached 12 (see Table S1). [file peerj-11-15991-s007.png]

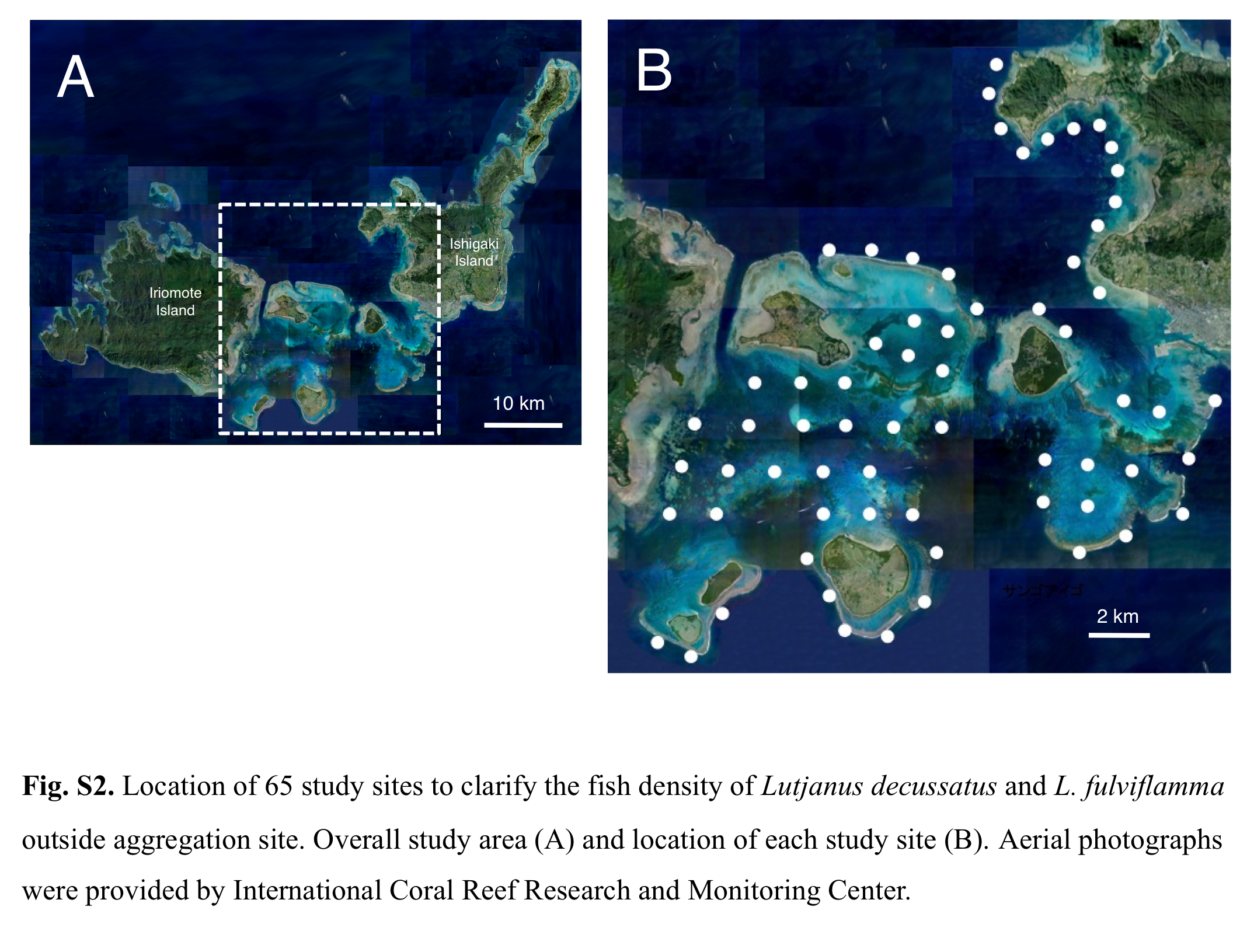

Supplement: Supplemental Information 8 — Overall study area (A) and location of each study site (B). Aerial photographs were provided by International Coral Reef Research and Monitoring Center. [file peerj-11-15991-s008.png]
